# Association Between the Neutrophil/Lymphocyte Ratio and Acute Kidney Injury After Cardiovascular Surgery: A Retrospective Observational Study

**DOI:** 10.1097/MD.0000000000001867

**Published:** 2015-10-30

**Authors:** Won Ho Kim, Ji Young Park, Seong-Ho Ok, Il-Woo Shin, Ju-Tae Sohn

**Affiliations:** From the Department of Anesthesiology and Pain Medicine, Samsung Changwon Hospital, Sungkyunkwan University School of Medicine, Changwon (WHK); Department of Anesthesiology and Pain Medicine, Gyeongsang National University Hospital (JYP, S-HO, I-WS, J-TS); Department of Anesthesiology and Pain Medicine, Gyeongsang National University School of Medicine (WHK, S-HO, I-WS, J-TS); and Institute of Health Sciences, Gyeongsang National University, Jinju, Republic of Korea (J-TS).

## Abstract

A high neutrophil-lymphocyte ratio (N/L ratio) was associated with the development of acute kidney injury (AKI) in patients with severe sepsis. We sought to investigate the association between the perioperative N/L ratios and postoperative AKI in patients undergoing high-risk cardiovascular surgery.

A retrospective medical chart review was performed of 590 patients who underwent cardiovascular surgeries, including coronary artery bypass, valve replacement, patch closure for atrial or ventricular septal defect and surgery on the thoracic aorta with cardiopulmonary bypass (CPB). Baseline perioperative clinical parameters, including N/L ratios measured before surgery, immediately after surgery, and on postoperative day (POD) one were obtained. Multivariate logistic regression analysis was used to evaluate risk factors.

A total of 166 patients (28.1%) developed AKI defined by the KDIGO (kidney disease improving global outcomes) criteria in the first 7 PODs. Independent risk factors for AKI included old age, decreased left ventricular systolic function, preoperative high serum creatinine, low serum albumin and high uric acid levels, intraoperative large transfusion amount, oliguria, hyperglycemia, and elevated N/L ratio measured immediately after surgery and on POD one. The quartiles of immediately postoperative N/L ratio were associated with graded increase in risk of AKI development (fourth quartile [N/L ratio≥10] multivariate odds ratio 5.90, 95% confidence interval [CI] 2.74–12.73; *P* < 0.001), a longer hospital stay, and a higher in-hospital and 1-year mortality rate (fourth quartile [N/L ratio≥10] adjusted hazard ratio for 1-year mortality [8.40, 95% CI 2.50–28.17]; *P* < 0.001).

In patients undergoing cardiovascular surgery with CPB, elevated N/L ratios in the immediately postoperative period and on POD one were associated with an increased risk of postoperative AKI and 1-year mortality. The N/L ratio, which is easily calculable from routine work-up, can therefore assist with risk stratification of AKI and mortality in high-risk surgical patients.

## Abstract 2

Supplemental Digital Content is available in the text

## INTRODUCTION

Acute kidney injury (AKI) after cardiovascular surgery is a serious complication and is associated with increased medical cost and substantial mortality.^1,2^ The incidence of AKI after cardiovascular surgery has been reported to be as high as 55% and the incidence of renal replacement therapy (RRT) to be 2% to 8%.^1,3–11^ Acute kidney injury is associated with up to 60% mortality rates in cardiac surgery patients,^12^ and the risk of death associated with AKI remains high for 10 years, even for those patients with complete renal recovery.^1^ As there is no effective therapy available for AKI after cardiovascular surgery,^13,14^ accurate prediction of AKI may provide an opportunity to develop strategies for early diagnosis and intervention to optimize outcomes.^10,15,16^

Previous studies have identified risk factors for AKI after cardiovascular surgery, and several risk-scoring models with independent risk factors have been developed to increase predictability.^4–11,17,18^ However, there is a discrepancy in risk factors identified in these studies, and a recent study questioned the predictability of previous risk scores by applying the gray zone approach.^19^ Recently, several promising plasma and urine biomarkers reflecting renal injury, including cystatin-C and interleukin-18, have been introduced to facilitate early diagnosis.^20,21^ However, these biomarkers are costly and not sufficiently validated, it is still necessary to develop a clinically useful and cost-effective risk factor of postoperative AKI.

The role of direct inflammatory injury in the pathogenesis of AKI is well recognized in addition to ischemia-reperfusion injury, endothelial cell dysfunction, and apoptosis.^22,23^ Recent clinical and laboratory studies are reporting that inflammation develops during ischemia-reperfusion injury and that AKI occurs along with the systemic inflammatory response.^22–24^ The neutrophil-lymphocyte ratio (N/L ratio), a surrogate marker for systemic inflammatory response, is inexpensive and can be easily calculated from a complete blood cell count with differential.^25^ The N/L ratio has been reported to be a predictor and prognostic marker of bacteremia in medical emergencies.^26,27^ Previous studies have also reported on the N/L ratio as a prognostic marker for various types of cancer.^28–31^ Furthermore, the N/L ratio can also predict the prognosis of percutaneous coronary intervention and coronary artery bypass graft (CABG).^32–35^ In previous studies, a high N/L ratio was associated with poor baseline renal function^33,34^ and served as an independent risk factor for AKI in patients with severe sepsis.^36^ However, the predictive utility of the N/L ratio for postoperative AKI has not previously been evaluated in patients undergoing cardiovascular surgery. We hypothesized that the N/L ratio could help predict AKI after cardiovascular surgery. The purpose of this study was to investigate whether a preoperative or postoperative N/L ratio could be an independent predictor of AKI, as well as clinical outcome in patients undergoing high-risk cardiovascular surgery.

## METHODS

After obtaining Samsung Changwon Hospital Institutional Review Board approval (2015-SCMC-011-00) and Gyeongsang National University Institutional Review Board approval (GNUH-2015-03-019-002), the electronic medical records were retrospectively reviewed in 600 consecutive adult patients who had undergone open cardiac or thoracic aorta surgery with cardiopulmonary bypass (CPB) at the reporting institution between 2009 and 2014. This retrospective observational study was registered at http://cris.nih.go.kr (KCT0001483). The surgeries included CABG, valve surgery, patch closure for atrial or ventricular septal defect, and thoracic aortic surgery (Supplemental Table 1, http://links.lww.com/MD/A503). The need for informed consent was waived for this study, given the retrospective design. Patients were excluded if they had missing preoperative serum creatinine (sCr) values (n = 3), missing preoperative or postoperative differential blood cell counts (n = 0), preoperative renal replacement therapy (RRT, n = 3), or if they died within 48 h postoperatively (n = 4). Of the remaining 590 patients, 166 (28.1%) developed AKI, as defined by to the KDIGO (Kidney Disease Improving Global Outcomes) criteria.^37^

Demographic or perioperative parameters previously known to be related to postoperative renal dysfunction were included in this study after literature review (Table 1) (Supplemental Table 1, http://links.lww.com/MD/A503).^3,7,8,10,11,38–44^ They included medical history, baseline cardiovascular status, surgery-related factors, anesthesia details, and blood test results. The differential cell counts were obtained and N/L ratios calculated at 3 time points: preoperative, immediately postoperative (within 1 h after arrival at ICU), and postoperative day (POD) one.

**TABLE 1:**
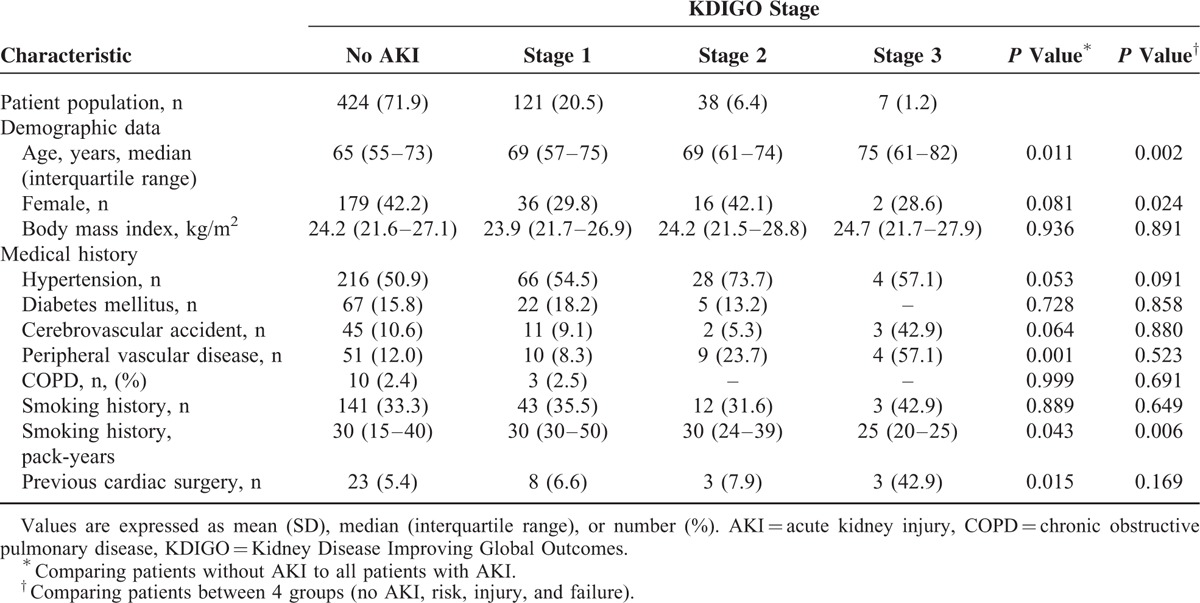
Baseline Patient Characteristics by the KDIGO Stage of Acute Kidney Injury

The development of AKI within the first postoperative week was the primary measured outcome. Again, AKI was defined according to the KDIGO criteria,^45^ which classify AKI by severity based on the maximal change in sCr from preoperative baseline levels. All patients who met the KDIGO criteria for stage 1, 2, and 3 were identified as having AKI. Renal replacement therapy was defined as a new need for dialysis after surgery. Postoperative outcome variables included the need for postoperative RRT, length of hospital and ICU stay, and in-hospital and 1-year mortality. The incidences of postoperative complications including pulmonary infection, cerebrovascular accident, and resternotomy due to postoperative bleeding were compared. The incidence of postoperative continuous RRT was also compared.

Anesthesia was maintained by total intravenous anesthesia. Arterial cannulation was performed in the right axillary artery, femoral artery or ascending aorta, and venous cannulations were bicaval or in the right appendage according to type of surgery. Cardiopulmonary bypass was routinely instituted at 2.2 to 2.5 L/min/m^2^. Aprotinin and tranexamic acid were not used for coagulation support.

SPSS software version 21.0 (IBM Corp, Armonk, NY) was used to analyze the data. For all analyses, *P* < 0.05 was considered statistically significant. A sample size of 400 patients or more was determined under the assumption that the expected odds ratio of AKI development in patients with increased N/L ratio would be 2.0, with a power of 0.8 and a type I error of 0.05.^46^ For accurate estimation, the sample size was also validated according to a target number of outcome events of 10 per independent predictor.^47^ For the present study, this was estimated to be 400 patients or more, thereby permitting unbiased accommodation of 10 or fewer predictive variables in a multiple logistic regression model (estimated 25% incidence of postoperative AKI).^47^

Categorical variables were reported as an absolute number (*n*) and a relative frequency (%) and continuous variables were reported as a median (interquartile range). Missing data except sCr was present in <1% of records. Missing values for categorical variables were assigned the most frequent gender-specific value, whereas continuous values were assigned gender-specific median values. Categorical variables were compared using Fisher's exact test or the χ^2^ test, according to expected counts. Continuous variables between those with and without AKI were compared using the unpaired *t* test or the Mann–Whitney *U* test, according to normality. Comparison of continuous variables among those without AKI and those with all 3 KDIGO stages was done using 1-way analysis of variance or the Kruskal–Wallis test. Logistic regression models were used to identify univariate and multivariate predictors for AKI. Univariate logistic regression analysis was used first to identify possible risk factors for AKI, with the multivariate model including only variables that were significant on univariate analysis (*P* < 0.05). Continuous variables were categorized before performing logistic regression analysis. The cut-off point was determined for continuous variables using the receiver operating characteristic (ROC) curve that had the maximal sum of sensitivity and specificity. N/L ratio variables were categorized by quartiles, with the lowest N/L ratio quartile used as a reference. Variables with commonly used normal values, such as left ventricular ejection fraction (LVEF), were categorized using their normal cut-off values. The cut-off levels for serum albumin and uric acid were determined according to previous studies.^39,40^ Predictor variables were selected from a list of candidate variables (Table 2) by performing a forward stepwise variable selection with a significance criterion of *P* < 0.05.

**TABLE 2:**
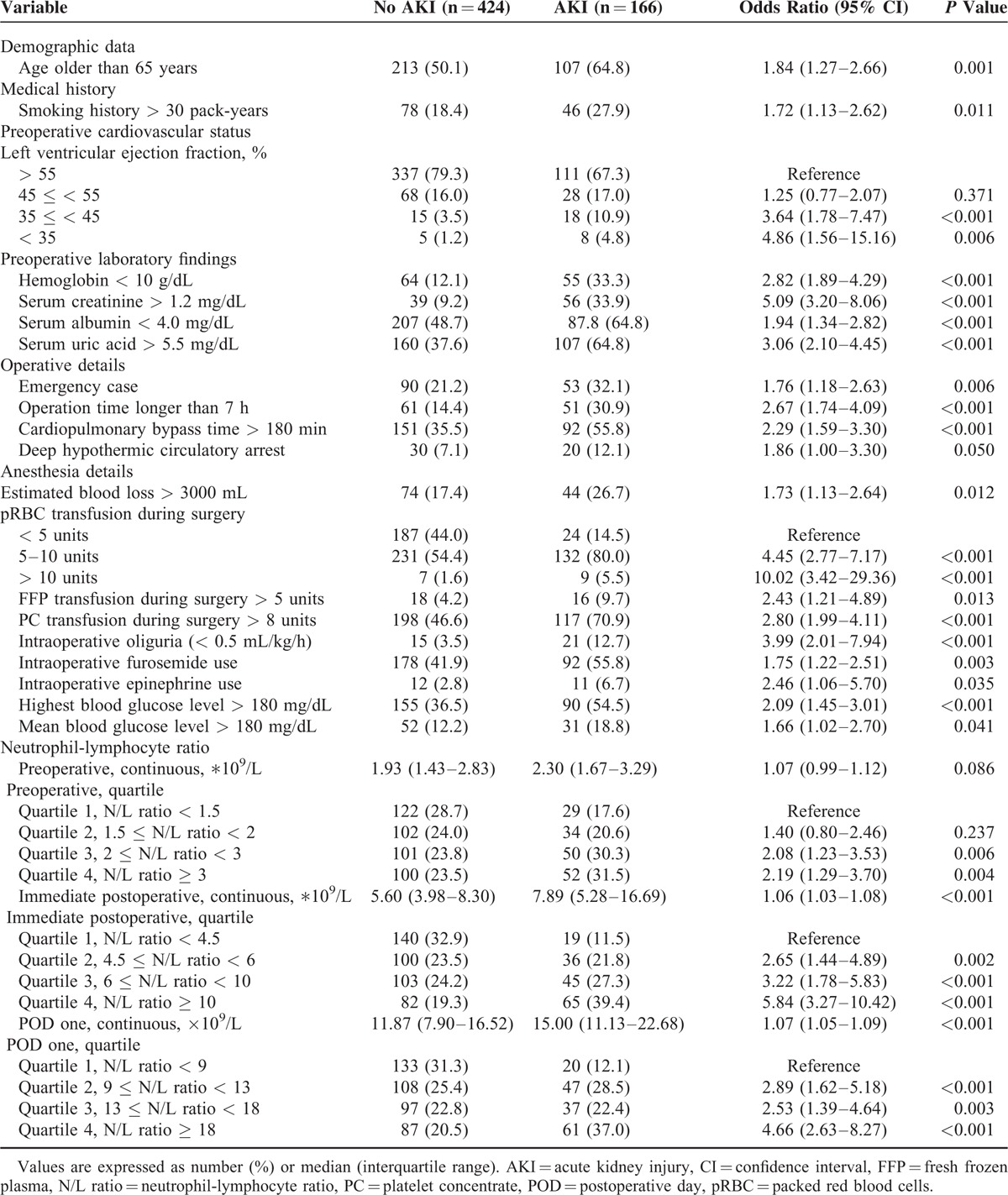
Univariate Logistic Regression Analysis of Categorized Risk Factors for Postoperative Acute Kidney Injury in All KDIGO Stages

Stepwise forward Cox proportional hazard regression models were used to identify the uni- and multivariate covariates associated with mortality. A Kaplan–Meier curve was used to plot survival in each of the 4 quartiles and a log rank test was used to compare survival across quartiles.

## RESULTS

Among patients who underwent cardiovascular surgery with CPB between 2009 and 2014 (n = 600), a total of 590 patients were analyzed after the exclusion of 10 patients. Of these 590 patients, 166 (28.1%) developed AKI as defined by the KDIGO criteria, and 35 (5.9%) required RRT within the first 7 postoperative days.

**FIGURE 1:**
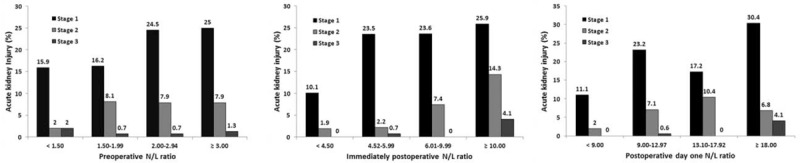
Association between the perioperative neutrophil-lymphocyte ratio (N/L ratio; preoperative, immediately postoperative, and postoperative day one) and incidence of acute kidney injury KDIGO stages after surgery with cardiopulmonary bypass. Vertical bars denote the proportions of AKI within categories defined by the perioperative N/L ratio. AKI = acute kidney injury, KDIGO = Kidney Disease Improving Global Outcomes, N/L ratio = neutrophil-lymphocyte ratio.

Demographic and perioperative parameters according to the grade of AKI in the whole study sample are presented in Table 1 and Supplemental Tables 1 and 3, http://links.lww.com/MD/A503. There were differences in demographics, medical history, preoperative cardiovascular status, and baseline laboratory findings between patients with and without AKI. The preoperative, immediately postoperative, and POD one N/L ratios were divided into quartiles, as shown in Table 2. The association between the N/L ratio at 3 time points and incidence of AKI is shown in Figure 1.

The results of both univariate and multivariate analyses of risk factors for AKI within all KDIGO stages are displayed in Tables 2 and 3. An elevated total white blood cell count (WCC), elevated segmented neutrophil count, and depressed lymphocyte count at certain time points were also associated with an increased postoperative AKI (Supplemental Table 2, http://links.lww.com/MD/A503). However, N/L ratios (POD one χ^2^ = 36.05) were stronger univariate predictors of AKI than the total WCC (POD one χ^2^ = 5.08), segmented neutrophil count (POD one χ^2^ = 2.44), or lymphocyte count (POD one χ^2^ = 12.11). Furthermore, multivariate logistic regression revealed that the immediately postoperative and POD one N/L ratios were independent predictors of AKI (Table 3).

**TABLE 3:**
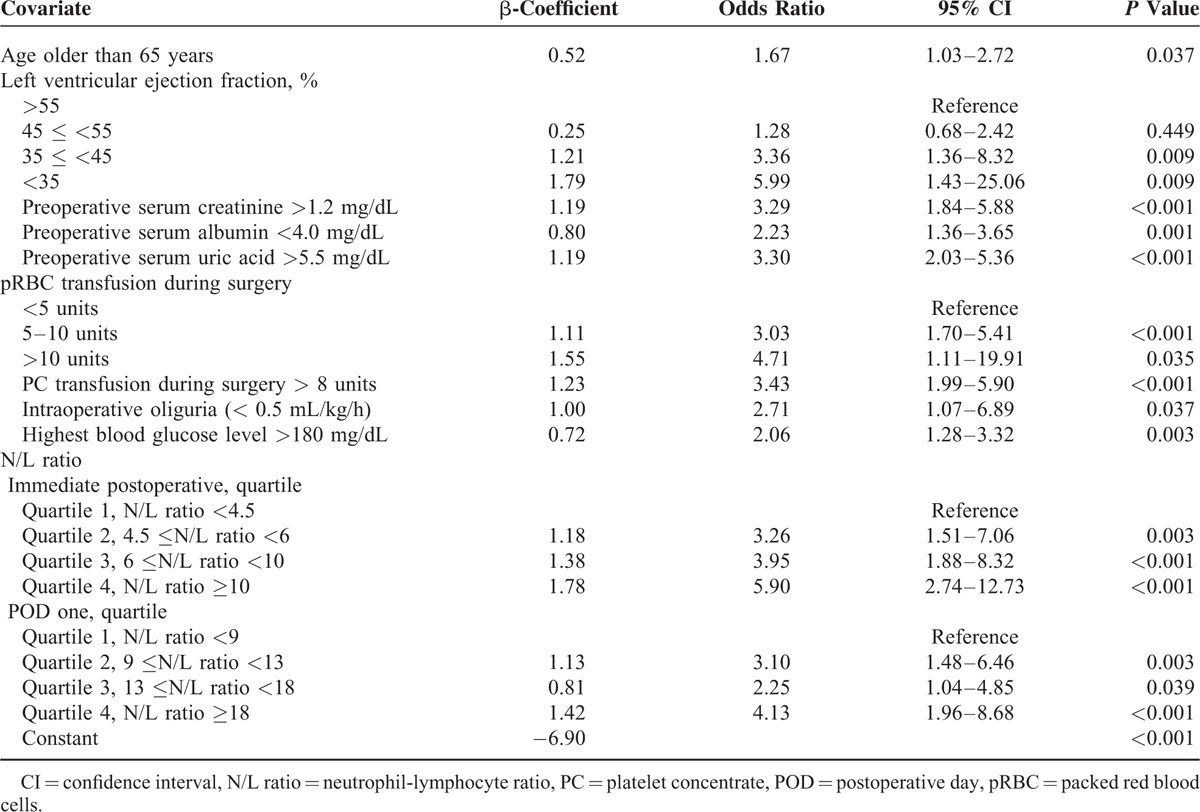
Multivariate Logistic Regression Analysis of Risk Factors for Postoperative Acute Kidney Injury in All KDIGO Stages

Among the potential risk factors evaluated by univariate analysis, independent risk factors for AKI included age >65 years, decreased LVEF (quartile), preoperative sCr >1.2 mg/dL, serum albumin < 4.0 mg/dL, serum uric acid >5.5 mg/dL, large pRBC transfusions (quartile), platelet concentrate transfusion >8 units, intraoperative oliguria, highest intraoperative blood glucose >180 mg/dL, and N/L ratio (quartile) immediately postoperative and on POD one. The quartiles of the immediately postoperative N/L ratio were associated with graded increases in the risk of AKI development (4th quartile [N/L ratio≥10] multivariate odds ratio [OR] 5.90, 95% confidence interval [CI] 2.74–12.73; *P* < 0.001) (Table 3).

Table 4 displays baseline characteristics and early postoperative outcomes according to the immediately postoperative N/L ratio quartiles. Patients with higher N/L ratios tended to have previous history of angina, poor cardiac function, longer CPB time, large volume of blood loss and large intraoperative transfusion of fresh frozen plasma, and platelet concentrate. They were also more likely to have postoperative pulmonary infections (*P* = 0.015). A relationship between the N/L ratio quartiles and length of hospital stay was observed (*P* < 0.001). Higher quartile were associated with increased in-hospital and 1-year mortality rates (4th quartile [N/L ratio≥10] adjusted hazard ratio [HR] for 1-year mortality 8.40, 95% CI 2.50–28.17]; *P* < 0.001). The independent predictors of mortality in a Cox proportional hazard model, which included all the variables in Table 2, are shown in Table 5. The immediately postoperative N/L ratio as a continuous variable remained as an independent predictor of mortality (HR 1.02 per unit, 95% CI 1.01–1.04, *P* = 0.006).

**TABLE 4:**
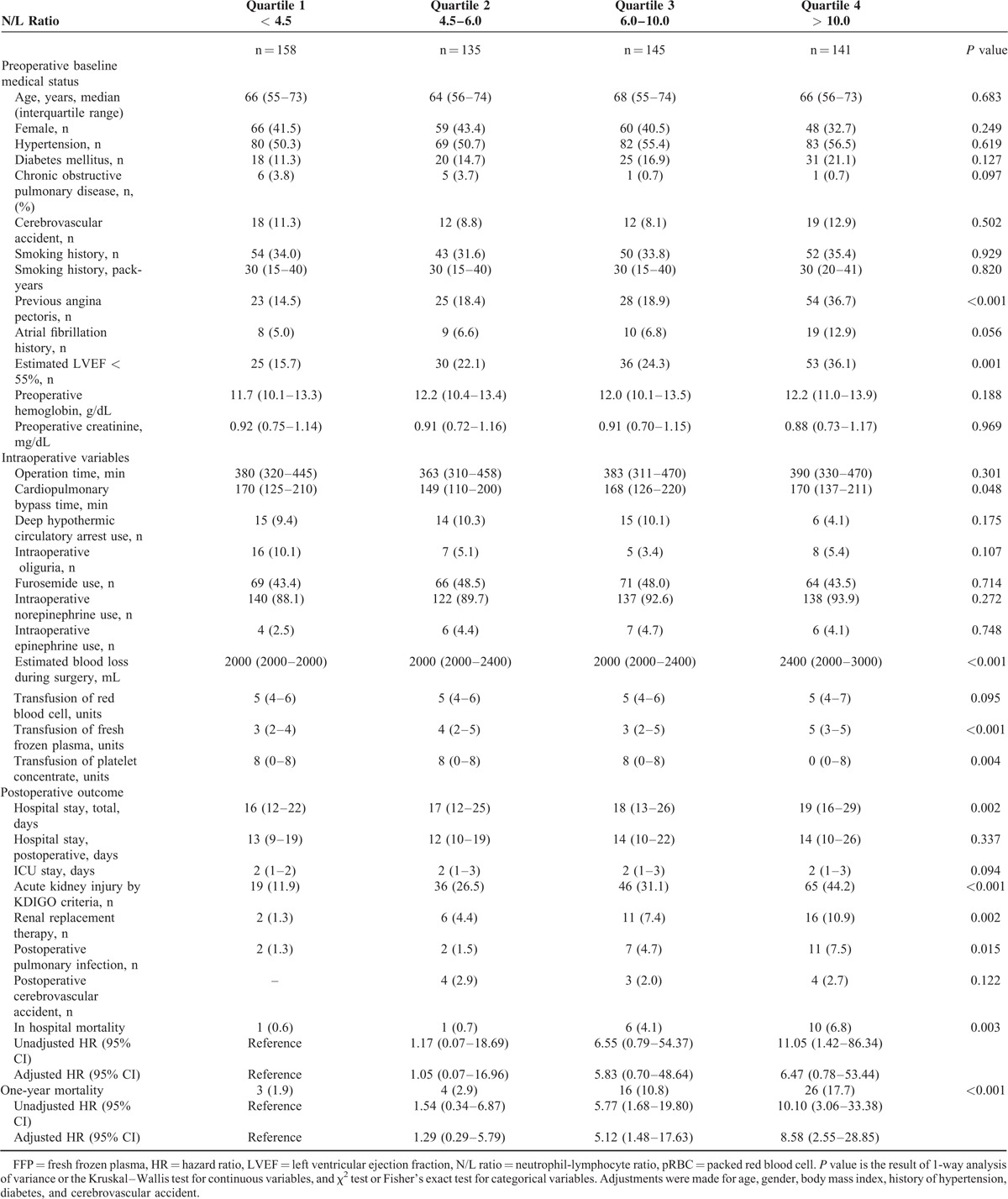
Baseline Patient Characteristics and Early Postoperative Outcomes According to the Immediately Postoperative N/L Ratio

**TABLE 5:**
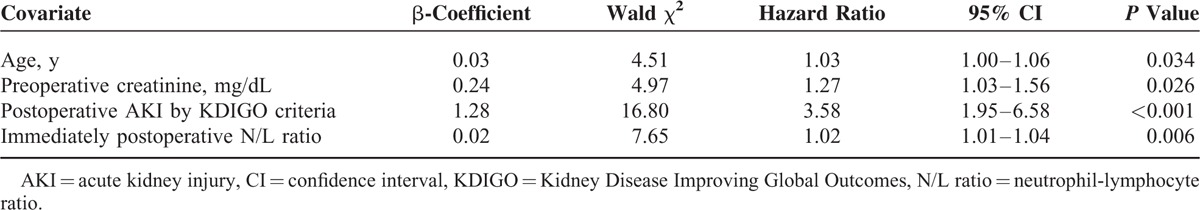
Multivariate Predictors of 1-Year Mortality

**FIGURE 2:**
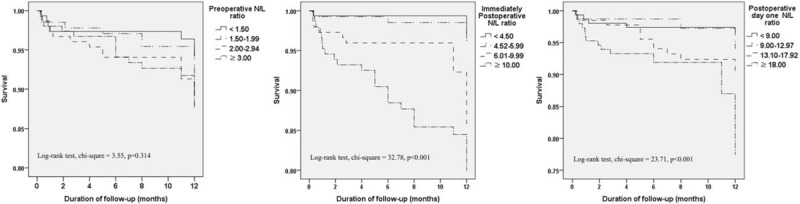
One-year survival of patients stratified by quartiles of neutrophil-lymphocyte ratios (N/L ratios) at 3 time points (preoperative, immediately postoperative, and postoperative day one). N/L ratio = neutrophil-lymphocyte ratio.

Figure 2 shows Kaplan–Meier curves displaying the relationship between all-cause mortality and the N/L ratio preoperative, immediately postoperative and on POD one. The mortality rate was significantly lower in immediately postoperative N/L ratios that fell in quartiles 1 and 2 than in ratios belonging to quartiles 3 and 4 (quartile 1 vs 3, χ^2^ = 10.01, *P* = 0.002; quartile 1 vs 4, χ^2^ = 22.10, *P* < 0.001, quartile 2 vs 3, χ^2^ = 6.55, *P* = 0.010; quartile 2 vs 4, χ^2^ = 16.35, *P* < 0.001). The mortality rate was significantly lower in POD one N/L ratios that fell in quartile 1 than those belonging to quartiles 3 and 4, and lower for quartile 2 than for quartile 4 (quartile 1 vs 3, χ^2^ = 4.06, *P* = 0.044; quartile 1 vs 4, χ^2^ = 16.02, *P* < 0.001, quartile 2 vs 4, χ^2^ = 13.22, *P* < 0.001).

## DISCUSSION

In this retrospective study, an elevated N/L ratio immediately postoperative or on POD one was associated with an increased risk of AKI development during the first postoperative week, whereas the preoperative N/L ratio did not predict risk. Furthermore, an elevated N/L ratio immediately postoperative was an independent predictor of 1-year mortality. Patients with postoperative AKI had higher N/L ratios than those without, and the quartiles of these N/L ratios were associated with graded increases in risk of postoperative AKI.

The association between the N/L ratio and postoperative AKI development can be traced to the role of inflammation in the pathogenesis of AKI.^22–24^ Ischemia/reperfusion injury and inflammation are suggested to play critical roles in the development of AKI.^22–24,48^ An acute ischemic insult activates endothelial renal cells that express adhesion molecules, thus facilitating adhesion of inflammatory blood cells.^48^ Furthermore, CPB can induce systemic inflammatory response syndrome.^48,49^ The total WCC, neutrophil count, and lymphocyte count are all potential surrogate marker of inflammation. In fact, the total WCC has been found to predict mortality after cardiac surgery.^50^ However, an association between leukocytosis and clinical outcome was not proven, and the ability of the WCC to predict postoperative AKI and mortality was weak in this study (Table 5) (Supplemental Table 1, http://links.lww.com/MD/A503). On the other hand, regarding white blood cell subtype, neutrophil and lymphocyte counts have been associated with the development of cardiovascular events.^51,52^ The predictive value of these 2 components can be combined by calculating the N/L ratio, which has been reported to be a prognostic marker for bacteremia, coronary intervention, CABG, and various types of cancer.^28–35^ We demonstrated that the N/L ratio is a stronger independent predictor of postoperative AKI and 1-year mortality than total WCC, neutrophil count, and lymphocyte count.

Although the preoperative N/L ratio quartile as a categorical variable was a significant predictor of postoperative AKI when analyzed by univariate logistic regression, it was not found to be an independent predictor by multivariate analysis. The preoperative N/L ratio as a continuous variable was not a significant predictor of postoperative AKI. Immediately postoperative and POD one N/L ratios were significant predictors of AKI in multivariate analysis both as continuous and categorized variables. This strong association between postoperative N/L ratios and AKI within the first postoperative week may be explained by the fact that the postoperative N/L ratios can reflect the inflammatory process during the surgery with cardiopulmonary bypass better than the preoperative N/L ratio can. Surgery induces inflammatory reactions, which are particularly prominent after cardiac surgery,^53^ and CPB in particular causes an inflammatory response by activating endothelial cells and neutrophils, as well as upregulating adhesion factors.^54^

Our study results confirm and extend previous reports on the prognostic role of the N/L ratio. As previously mentioned, the N/L ratio and lymphopenia can predict bacteremia in the emergency care unit and the N/L ratio is also an independent predictor of mortality in patients with bacteremia.^26,27^ Further, the N/L ratio has been reported to be a prognostic marker of colorectal, gastric, and lung cancer, as well as in patients undergoing percutaneous coronary intervention and CABG.^28–35^ However, few studies have assessed the relationship between the postoperative N/L ratio and postoperative AKI. A high preoperative N/L ratio was associated with poor baseline renal function in patients undergoing CABG^33,34^ and was also an independent predictor of AKI in patients with severe sepsis.^36^

The results of our study revealed risk factors for cardiac surgery-associated AKI that are mostly consistent with previous studies. Previous studies have reported that old age,^3^ decreased LVEF,^3,10,11^ poor baseline renal function,^7,8,10^ hypoalbuminemia,^38,39,41^ hyperuricemia,^40^ intraoperative large volume transfusion,^8^ intraoperative oliguria,^42^ and high intraoperative blood glucose levels ^43,44^ are associated with postoperative AKI.

Our risk factors including the N/L ratio can help physicians plan postoperative monitoring and management based on a patient's risk of AKI. High-risk patients can be monitored with biomarkers of AKI.^20,21^ Additionally, although there is still paucity of evidence, RRT can be commenced early^15,16^ or anti-inflammatory therapy can be applied.^48^ Nephrotoxic combination of nonsteroidal anti-inflammatory drugs with renin-angiotensin system inhibitors and/or diuretics should be avoided.^55^ Our risk factors can also improve selection of high-risk patients by incorporation into inclusion criteria of clinical trials.

This study had several limitations. First, due to a retrospective design, our results can only suggest an association between the N/L ratio and postoperative AKI. It was difficult to control for bias and confounders, despite conduction of multivariate analysis and covariate adjustments. Prospective validation of the N/L ratio is required. Second, a relatively small number of patients was reviewed compared to previous studies.^32,33^ As this study was powered to identify a potential risk factor for AKI and the sample size was determined based on an assumed odds ratio, the analysis of mortality is of limited value. Third, as this study was performed in only 2 institutions, external validity is limited. Fourth, the patients included in this study had undergone any of 4 different surgeries, which may have confounded data analysis. However, only surgeries with CPB were included and the immediately postoperative N/L ratio was determined to be a significant predictor of postoperative AKI in our subgroup analysis of CABG, valve replacement, and thoracic aortic surgery.

In conclusion, this study demonstrated a robust and independent association between immediately postoperative and POD one N/L ratios and postoperative AKI in the first 7 days following cardiovascular surgery with CPB. Furthermore, an elevated postoperative N/L ratio was associated with an increased 1-year mortality rate. The N/L ratio, which is easily calculated and routinely available, can therefore assist in identifying patients at risk for AKI and predict poor survival in high-risk surgical patients.
